# Advancing Sports Injury Treatment Through Cell-Free MSC-Based Therapies: A Narrative Review

**DOI:** 10.3390/muscles5030056

**Published:** 2026-08-06

**Authors:** Duaa Abuarqoub, Alqassem H. Abuarqoub, Mais Emad, Majd F. Ellauzi

**Affiliations:** 1 Faculty of Pharmacy and Medical Sciences, University of Petra, Amman 11196, Jordan; mais999@hotmail.co.uk; 2 Department of Biotechnology, Faculty of Allied Medical Sciences, Al-Ahliyya Amman University, Amman 19111, Jordan; aha87@georgetown.edu; 3 Al Wafi Academy, Al-Wafi Group, Amman 11190, Jordan; majd@al-wafigroup.com

**Keywords:** stem cells, extracellular vesicles (EVs), conditioned medium (CM), sport medicine, regenerative medicine

## Abstract

Background: Sports-related musculoskeletal injuries, including tendon, ligament, muscle, and cartilage damage, often heal slowly and incompletely. Current treatments may not fully restore tissue function, necessitating regenerative therapies. Objective: This study was conducted to review the therapeutic potential of stem cell-derived extracellular vesicles (EVs) and conditioned medium (CM) as cell-free approaches to treating musculoskeletal injuries in sports medicine. Methods: A narrative review of preclinical and early clinical studies investigating the biological mechanisms and regenerative effects of mesenchymal stem/stromal cell (MSC)-derived EVs and CMs published between 2019 and 2026 was performed by searching Scopus, PubMed, Web of Science, and Google scholar. Results: EVs and CMs contain bioactive molecules, including microRNAs, cytokines, and growth factors, that modulate inflammation, promote tissue repair, enhance angiogenesis, and support extracellular matrix remodeling. Preclinical studies demonstrate improved healing of tendons, ligaments, muscles, and cartilage, while early clinical evidence suggests favorable safety and promising therapeutic outcomes. However, the variety of MSC sources, EV separation methods, characterization procedures, and treatment protocols present major challenges in determining the reliability and therapeutic efficacy of these therapies. Notably, the number of clinical studies on EV and CMs is still limited, and most of the available studies are in vitro studies. Conclusions: MSC-derived EVs and CMs represent promising cell-free regenerative therapies for sports-related musculoskeletal injuries. The available evidence, which demonstrates their ability to enhance healing while avoiding the challenges associated with cell-based therapies, highlights their translational potential. However, most of the studies that are now available are preclinical or early-stage clinical studies, and there is still a limited number of clinical trials. Further well-designed clinical studies are needed to establish standardized protocols and confirm long-term safety.

## 1. Introduction

Athletes of all skill levels and ages frequently experience sports injuries, which often result in lengthy recovery times, lower athletic performance, and a lower quality of life [1,2]. According to a recent retrospective analysis of almost 8000 Olympic participants, the knee and shoulder joints are most vulnerable to serious injuries [3]. Correspondingly, according to a 22-year registry study of Swedish Olympic athletes, the knee and lower limbs were injured the most frequently, with an average of one injury per two years for each participant. Males and females had comparable injury rates, with high-impact sports having the greatest rates [4]. Many sports injuries respond well to conventional therapies, like rest, ice, compression, elevation, courses of anti-inflammatory and pain-killer medications, physical therapy, physiotherapy, and surgery [5]. Nevertheless, these treatments are not always successful and can result in long-term issues, like early-onset osteoarthritis (OA), and contribute to public health expenses. This is due to the recognized poor vascular supply of tendons and ligaments, which consequently slows down healing processes and extends the time to full recovery [5,6]. Additionally, injuries not only limit physical activity but can also dampen an athlete’s spirit, as athletes find delight and rejuvenation in sports. These challenges arising from sports injuries have led to an increased interest in regenerative medicine techniques that support genuine tissue healing in addition to symptom relief [7]. Recently, cell therapy has emerged as a promising approach in therapeutic applications and regenerative medicine. In sports medicine, stem cell therapy has been employed to treat a variety of injuries. Sports-related injuries frequently cause tissue damage to ligaments, tendons, cartilage, muscles, and bones [5]. Stem cell therapy has the potential to treat various injuries by promoting tissue repair and regeneration, accelerating recovery, and restoring function [8]. In sports medicine, mesenchymal stem cells (MSCs) are one of the most studied cell types. Through osteogenic, myogenic, and chondrogenic differentiation, MSCs have the ability to heal tendon, ligament, cartilage, and muscle damage [9]. In sports medicine, stem cells and their secreted products—such as conditioned media and extracellular vesicles (EVs) such as exosomes—are thoroughly investigated for their regenerative potential in musculoskeletal injuries [10].

Several reviews have described the biological features and therapeutic potential of MSC-derived EVs and secretome-based therapies; however, most of these reviews focused on regenerative medicine or a specific tissue and conditioned media (CM)-based therapeutics. Nevertheless, the majority focused on regenerative medicine in general. Remarkably, a complete examination of cell-free therapy in the context of sports-related musculoskeletal injuries, incorporating both preclinical and currently emerging clinical evidence while considering the challenges and limitations, is still lacking. Although the primary focus of this review is the examination of MSC-derived CMs and EVs, highlighting their mechanism of action, therapeutic outcomes, existing limits, and challenges to successful clinical applications in sports medicine, selected studies involving broader musculoskeletal conditions, particularly osteoarthritis, were included when their findings were directly relevant to regenerative approaches used in sports medicine and musculoskeletal regeneration. This broader theory focuses on the molecular and clinical similarities between sports-related tissue damage, post-traumatic degeneration, and other musculoskeletal diseases that might impair athletic performance and rehabilitation. As a result, the scope of this review includes regenerative treatments for sports-associated musculoskeletal injuries, as well as closely linked musculoskeletal disorders with clinically significant evidence for tissue repair and functional recovery.

The main aim of the current manuscript is to summarize and evaluate the available studies on the therapeutic potential of using SCs, MSC-EVs, and CMs as regenerative therapies for sports-related musculoskeletal injuries.

## 2. Literature Search Strategy

This study was carried out as a narrative review in January 2026; to improve transparency and thoroughness, a structured literature search was carried out. The literature search period of this review was from 2019–2026, as most of the relevant studies were published in this period. Studies were found by searching electronic databases such as Scopus, PubMed, Web of Science, and Google Scholar.

The following keywords were among the search terms: stem cells, mesenchymal stem cells, extracellular vesicles, exosomes, conditioned medium, secretome, sports medicine, tendon injury, ligament injury, cartilage repair, muscle injury, and osteoarthritis. The literature selection process was independently performed by two reviewers. The analyzed studies were assessed for relevance to the subject of this narrative review, which included MSC-derived cell-free therapy for musculoskeletal injuries. Any conflicts regarding study selection were handled through discussion and agreement between the reviewers. Additionally, this review did not follow PRISMA guidelines, no protocol was registered, and no formal risk-of-bias assessment was performed.

**Figure 1:**
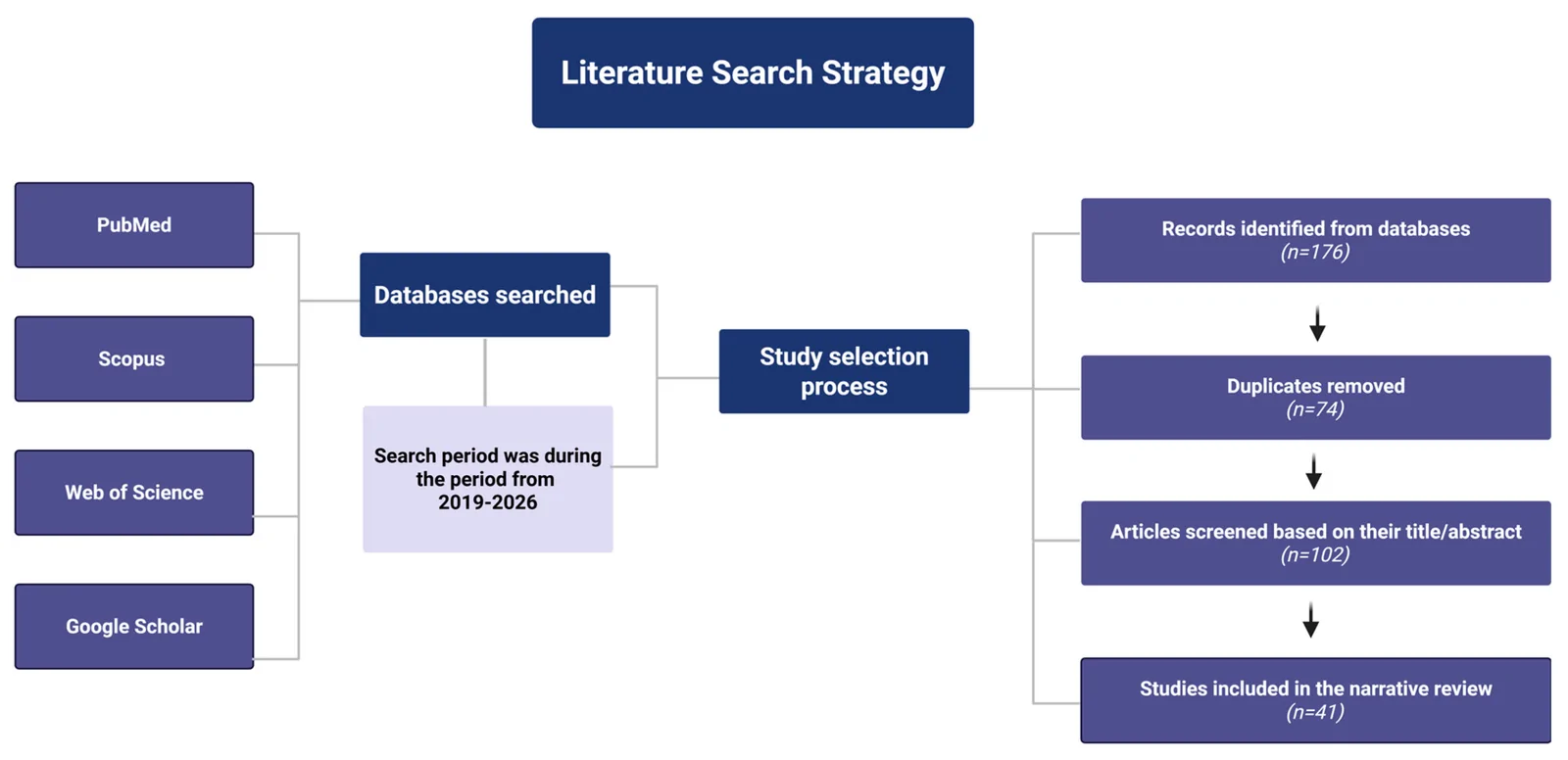
Literature search strategy flow diagram. This flow diagram represents the database searched, the search period, and the selection criteria of this narrative review.

The flow diagram in Figure 1 illustrates the literature search strategy.

### 2.1. Inclusion and Exclusion Criteria

Inclusion criteria: Preclinical, clinical, and translational studies evaluating MSC-derived extracellular vesicles or conditioned media for musculoskeletal regeneration and sports-related injuries were all eligible and included in the review.

Exclusion criteria: Studies that only addressed cell-based treatments, non-musculoskeletal uses, non-regenerative studies, conference abstracts, editorials, non-English publications, and articles with insufficient methodological information.

Screening of relevant references from selected articles was also conducted to identify additional studies.

### 2.2. Study Selection Process

The initial search of electronic databases such as Scopus, PubMed, Web of Science, and Google Scholar retrieved 176 records. After deleting 74 duplicates, 102 publications were screened based on their titles and abstracts. Following the full-text examination, 41 studies met the inclusion criteria and were included in the final analysis. Duplicate records of this narrative review were identified and removed during screening using EndNote software. Further, studies that did not directly address sports-related musculoskeletal injuries; studies that did not examine stem cells, EVs derived from MSCs, or CM; studies lacking relevant therapeutic outcomes; and publications that failed to meet the predetermined study-type or language criteria were excluded from this review. The final narrative review comprised the remaining 41 studies.

This review was conducted as a narrative review and did not follow a formal systematic review protocol or PRISMA methodology, despite the fact that a structured literature search and study-selection process were employed to improve transparency in obtaining relevant data. Therefore, rather than taking a systematic review approach, the structured search was designed to improve transparency in the literature identification process.

The flow diagram in Figure 1 illustrates the literature identification and selection procedure.

### 2.3. Assessment of Evidence Level

This paper presents a narrative review without a formal risk-of-bias assessment. However, the included studies were classified based on their level of evidence according to study design. In vitro studies were classified as preliminary evidence, animal studies as preclinical evidence, and clinical studies as having the highest level of proof. The interpretation of therapeutic efficacy took into consideration differences in study design, methodological approaches, and translational importance. The review included 41 references, which encompassed 10 preclinical studies, 6 clinical studies/clinical trials, 16 basic mechanistic studies, and 9 background, epidemiological, or review papers.

## 3. Stem Cell-Derived Products and Sports Medicine

Stem cell-derived product therapies employ paracrine signaling to deliver growth factors, cytokines, and signaling molecules that suppress inflammation, stimulate angiogenesis, improve cell migration and proliferation, and promote tissue repair and regeneration in damaged ligaments, tendons, cartilage, muscle, and bone [11]. For instance, stem cell-derived exosomes, a type of extracellular vesicles derived from stem cells (SC-EVs), show good prospects for soft tissue regeneration, cartilage repair, and immunological regulation. They are less immunogenic and easier to store than live cell transplants [12].

### 3.1. The Mechanism of Reactions in Sports Injuries by Stem Cell-Derived Products

**Figure 2:**
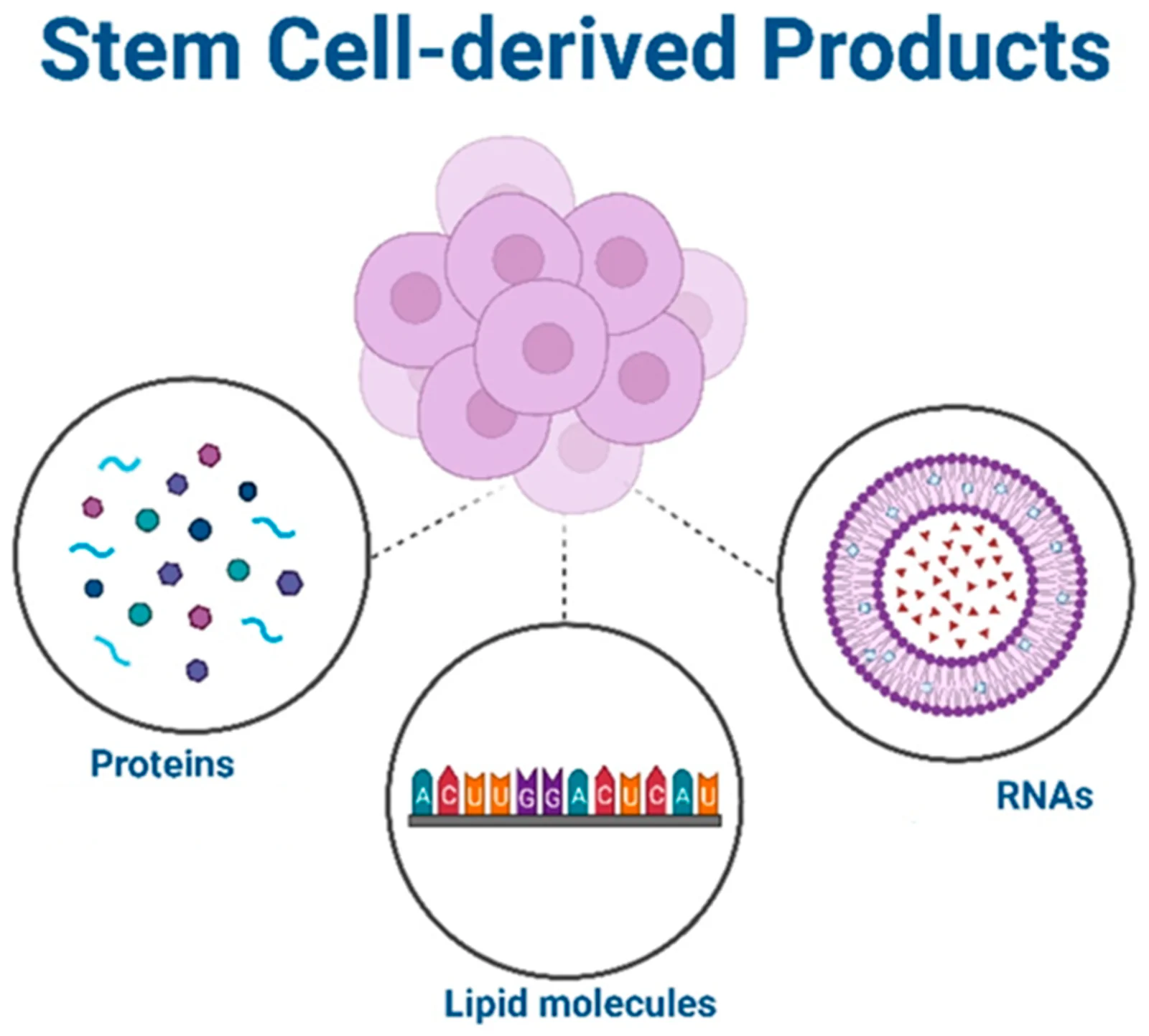
Stem cell-derived products are rich in proteins, lipid molecules, and RNAs, giving them great potential and promising applications in regenerative and sports medicine.

Stem cell-derived products possess great potential in enhancing tissue repair, suppressing inflammation, and promoting regeneration in musculoskeletal injuries [13]. They also contain a vast number of proteins, RNAs, and lipid molecules, which contribute to intercellular communication; see Figure 2 [14]. The composition and abundance of these components differ considerably depending on the cellular source, culture conditions, isolation methods, and analytical approaches employed. Stem cell-derived products’ miRNAs can be transferred to recipient cells, influencing their function and enabling the host cell to create various miRNAs in response to tissue repair [15,16]. Stem cell-derived products transport not only miRNAs but also over 1000 proteins, including enzymes, signaling molecules, functional lipids, and lipid-related enzymes. These components are required for the products’ stability, absorption, and function, and they play important roles in cellular communication, tissue healing, and inflammatory control; however, the specific effects of exosomes’ lipid contents are still unknown [17,18,19].

Stem cell-derived products affect the immune system through a variety of approaches: they reduce immune system overactivity by subverting M1 macrophage activity to regulatory M2 macrophages, lowering inflammatory CD4+ Th1 and Th17 lymphocyte production, decreasing dendritic cell (DC) antigen-presenting capacity, and inducing immunosuppressive Treg cells and tolerogenic DCs [20,21,22]. This ability to control immune cells, particularly macrophages, remains a primary focus of therapeutic development, as confirmed by recent studies [23]. Stem cell-derived products reduce inflammation by lowering pro-inflammatory cytokines like TNF-α, IL-1β, and IL-6 while increasing anti-inflammatory mediators like Arginase-1 [24]. Their therapeutic actions are primarily mediated by associated miRNAs (such as miR-21, miR-223, miR-181c, and miR-34a-5p), as well as immunomodulatory lipids and metabolites, which function synergistically to promote healing from inflammation and tissue repair [19,25].

### 3.2. Stem Cell-Derived Conditioned Media (SC-CM) in Sports Medicine

SC-CM, also sometimes called the secretome, is the culture supernatant collected from cultured stem cells and then centrifuged. The secretome, specifically MSCs, comprises a complex mix of secreted bioactive molecules such as cytokines, growth factors, chemokines, and extracellular vesicles. These secreted materials may have therapeutic effects by regulating inflammation, enhancing cell proliferation and migration, and facilitating tissue regeneration via paracrine signaling processes rather than direct cell replacement. Preclinical research indicates that SC-CMs can reduce pro-inflammatory cytokines and improve healing responses in wounded tissues, making it a promising cell-free therapy for regenerative medicine and sports medicine applications [26].

In an effort to investigate SC-CM, Soukup et al. studied the therapeutic benefits of MSC-CMs on inflammatory tendon cells. The conditioned media were produced from bone marrow-derived mesenchymal stromal cells (BM-MSCs) grown under controlled conditions. Following MSC expansion, the culture supernatant containing secreted bioactive components was collected and used for applications in sports medicine. Human tenocytes were exposed to a pro-inflammatory condition using IL-1β and TNF-α to mimic inflammatory tendon disease. Treatment with MSC-CMs demonstrated a possible role in significantly reduced inflammatory responses in tenocytes, as evidenced by lower expression of key inflammatory mediators such as IL-6, IL-8, COX-2, and matrix metalloproteinases (MMPs), all of which are involved in tendon degeneration and extracellular matrix breakdown. MSC-CMs may also help preserve the tenocyte phenotype and maintain the expression of genes involved in tendon homeostasis and matrix integrity. This study shows that MSC-CMs demonstrate an anti-inflammatory and cytoprotective effects on inflamed tenocytes, confirming the theory that MSC paracrine factors exhibit a potential in decreasing tendon inflammation and promote a regenerative microenvironment [10].

Additionally, researchers conducted an in vitro study [27] to investigate the impact of CMs from human umbilical cord-derived mesenchymal stem cells (UC-MSCs) on tenocytes from degenerative rotator cuff injuries that had been stimulated with interleukin-1β (IL-1β). The stem cells were isolated from healthy full-term umbilical cords and cultured; a number were exposed to synovial fluid from shoulders with rotator cuff tears to mimic the inflammatory joint conditions and increase bioactive factor secretion. The CM from synovial fluid-stimulated UC-MSCs (SF-UC-MSC CM) were mixed 50% with culture medium and applied to tenocytes treated with IL-1β to simulate tendinopathy [27].

In treated tenocytes, the CM demonstrated a significant modulation of inflammatory and matrix-related gene expression. It downregulated pro-inflammatory cytokines (IL-1β, TNF-α, IL-6, COX-2) and decreased PGE2 secretion, while increasing the expression of anti-inflammatory and tendon-healing genes (TIMP-1, TIMP-3, TGF-β1/2/3, IGF-1, bFGF). CM also affected extracellular matrix dynamics by increasing type I collagen gene expression and protein synthesis (thus enhancing the type I/III collagen ratio) while decreasing matrix-degrading enzyme expression. The CM effectively reversed IL-1β-induced mortality and apoptosis in tenocytes while maintaining viability. Overall, in cell culture, preclinical data revealed that the SF-UC-MSC secretome exhibits anti-inflammatory and cytoprotective properties on inflamed tenocytes, indicating potential for tendon healing applications in tendinopathy and sports medicine [27].

Moreover, Chamberlain et al., assessed the immunomodulatory effects in vitro and the therapeutic potential in vivo of CM from horse amniotic membrane-derived multipotent progenitor cells (AMCs), a type of mesenchymal-like stem/progenitor cell isolated from the horse’s amniotic membrane [28]. The in vitro study showed that AMCs can suppress the proliferation of activated peripheral blood mononuclear cells (PBMCs), both through direct contact and when separated in transwell systems, suggesting that soluble factors in the CM alone (AMC-CM) mediate immunomodulation. AMC-CM suppressed PBMC proliferation, suggesting the secretome contains anti-inflammatory signals. In pre-clinical application, 13 horses with spontaneously occurring tendon or ligament injuries received ultrasound-guided intralesional injections of the AMC-CM. No adverse effects, abnormal tissue development, or malignancies were found. Clinical and ultrasonographic follow-up revealed satisfactory healing results with a significantly decreased reinjury rate (~15.38%) compared to untreated instances. The conclusion was that AMC-derived CM can be considered a safe, cell-free therapeutic agent with potential to modulating immune responses and improve structural and functional healing in equine tendon and ligament injuries. This preclinical evidence highlights its potential use in regenerative medicine for musculoskeletal injuries [28].

Furthermore, although this review focuses on sports-related musculoskeletal injuries, many of the current clinical trials on MSC-derived EVs and CMs have been conducted in patients with knee osteoarthritis. Osteoarthritis (OA) is extremely important in sports medicine as previous joint trauma, ligament injuries, meniscal tears, and repetitive mechanical loading associated with athletic involvement are all known risk factors for the development of post-traumatic osteoarthritis [5,6]. Moreover, sports injuries and osteoarthritis have similar pathogenic processes, including inflammation, cartilage degradation, extracellular matrix remodeling, and impaired tissue repair [29]. Remarkably, evidence from osteoarthritis research provides insight into the suggested therapeutic potential, safety, and clinical application of cell-free treatments for sports-related musculoskeletal disorders.

Thus, the therapeutic potential of CM-MSCs to control the progression of OA was evaluated by Chen and his group. The MSC-CM was derived from cultured MSCs, and an OA animal model was developed in rats by surgically inducing joint instability through anterior cruciate ligament transection and medial meniscus destabilization [29]. Rats were divided into groups that received weekly intra-articular injections of MSC-CM or control injections of phosphate-buffered saline (PBS), with a separate sham-operated normal group. After 8 weeks, joints were evaluated using gross examination, histology, micro-CT imaging of subchondral bone, immunohistochemistry, and gene expression analysis. The MSC-CM group had a significantly preserved subchondral bone microarchitecture, a higher abundance of cartilage matrix components (type II collagen and aggrecan), and a more balanced extracellular matrix homeostasis, as evidenced by a lower ratio of matrix-degrading enzyme MMP-13 to its inhibitor TIMP-1 compared to PBS controls. Likewise, the CM suppressed chondrocyte apoptosis while enhancing autophagy marker expression (Beclin-1, LC3), indicating improved cellular viability processes. These combined results of animal studies, which demonstrated preclinical efficacy, showed that the MSC secretome can suggestively eliminate the development of OA by conserving bone structure, maintaining cartilage matrix balance, and activating autophagic processes in joint tissues, indicating that it has potential as a cell-free regenerative therapy for osteoarthritis and sports injuries [29].

Moreover, a preclinical study [30] investigated whether a CM derived from hepatocyte growth factor (HGF)-stimulated tendon stem cells (TSCs) could improve recovery in injured Achilles tendons. The stem cells employed were derived from rat Achilles tendons, which have mesenchymal stem cell-like features; the study focused on the whole CM. TSCs were treated with HGF to enhance their paracrine activity, and the resulting CM was examined using proteomic analysis, revealing an intricate array of repair-related soluble factors, with HGF functioning as a key regulatory node. In the in vitro experiment, the CM greatly increased fibroblast viability and migration, indicating improvement in cellular activity, which accelerates the healing process. For the preclinical work, a rat Achilles tendon injury model was treated with local CM injections, followed by histological, immunohistochemical, and biomechanical tests. In comparison to the untreated controls, the treated tendon groups showed an improvement in collagen fiber alignment, a reduction in inflammatory signaling cytokines (lower IL-6 and higher IL-10), enhanced extracellular matrix remodeling, and a significant increase in maximum tensile strength, though mechanical uniformity was not fully achieved. Inclusively, this work, which investigated preclinical efficacy in rat models, demonstrated that the HGF-induced TSC secretome appears promising in the enhancement of the tendon healing rate via paracrine and cell-free pathways, suggesting its promising applications in sports medicine in treating tendon injuries [30]. Studies on MSC-derived CM vesicles (EVs) show a promising regenerative potential in a variety of musculoskeletal injury models; however, the significance and mechanisms of these effects differ depending on the therapeutic product, MSC source, and experimental setting. For instance, Soukup et al. observed that a complete MSC-CM produced more potent anti-inflammatory effects in tenocytes than the isolated EV fraction, implying that CM activity may be due to the combined effect of EVs and soluble substances. Similarly, Lee et al. demonstrated that UC- MSC-derived CM suppressed inflammatory responses in degenerative rotator cuff tenocytes, while Chen et al. and Zhang et al. reported beneficial effects of an MSC-derived CM in osteoarthritis progression and Achilles tendon healing, respectively, emphasizing the influence of tissue source and injury model on therapeutic outcome.

Likewise, EV-based studies have also demonstrated promising regenerative effects, although differences between models remain evident [10,27,29,30]. (ADSC-derived EVs) reported improved tendon healing by modulating inflammation and tissue repair, whereas Chamberlain et al. concluded that MSC-derived exosomes and exosome-educated macrophages improved ligament healing, suggesting that EV-mediated immunomodulation may also contribute to repair in other musculoskeletal tissues. Collectively, these data suggest the therapeutic potential of MSC-derived cell-free products; nevertheless, differences in MSC source, EV/CM characterization, and experimental design limit direct comparison between studies, thereby emphasizing the need for standardized methodologies. 

Despite the promising results of these preclinical studies, variability in MSC source, donor features, expansion conditions, EV isolation, characterization, storage, delivery route, dosing and manufacturing procedures continues to limit the clinical translation of the EV/CM therapies. Table 1 summarizes these variability aspects, their variation across studies, and their potential impact.

#### SC-CMs in Clinical Trials

A clinical trial identified as NCT05777213 involved 27 participants and aimed to investigate the safety and wound-healing effects of conditioned medium obtained from human Wharton’s Jelly-derived mesenchymal stem cells (CM-WJMSCs) in patients suffering from trophic ulcers. Human umbilical cord Wharton’s Jelly is a rich source of MSCs that secrete a range of growth factors and cytokines into their growth media. The 27 participants in the study received intracutaneous injections of CM directly into the wound area, administering 0.1 cc per cm of wound at a frequency of two injections per month. The method of assessing wound healing was validated on a weekly basis by measuring changes in wound length, width, and area using digital measurements. The primary findings indicated a quantitative reduction in wound size over time, as well as the regeneration of granulated tissue and a decrease in edema and erythema. There was also thorough monitoring of any side effects or adverse events related to the CM injections. Notably, this clinical study represents one of the first efforts to provide evidence that the secretome of Wharton’s Jelly mesenchymal stem cells (MSCs) can promote tissue repair and modulate cytokines and growth factors in a therapeutic context for wound healing [31].

A Phase I/II randomized clinical safety trial (NCT07157891), sponsored by the University of Punjab, is currently underway (status: not yet recruiting). This study aims to evaluate the safety, feasibility, and early regenerative potential of the secretome derived from mesenchymal stem cells (MSCs). The secretome may be administered alone or in combination with Plasma Rich in Growth Factors (PRGF) through intra-articular injection in patients with mild to moderate knee osteoarthritis (KOA). The secretome is derived from human MSC cultures, specifically from adipose-derived MSCs (AD-MSCs) and umbilical cord-derived MSCs (UC-MSCs), which are grown under Good Manufacturing Practice (GMP)-compliant conditions. It contains a complex mixture of paracrine factors, including cytokines, growth factors, extracellular vesicles (such as exosomes), and various other bioactive molecules. In this open-label study, approximately 25 adult patients aged 30 to 55 with a diagnosis of Kellgren–Lawrence grade II-III knee osteoarthritis (KOA) will be randomly assigned to one of two groups. One group will receive 2 mL of an intra-articular secretome injection, administered twice with a 6-month interval between injections. The other group will receive a combination of secretome (2 mL) and autologous Platelet-Rich Growth Factor (PRGF) (1 mL), administered on the same schedule. PRGF is derived from each participant’s own blood and is activated to release growth factors before being mixed with the secretome. The primary outcomes of the study include pain reduction, measured using the Visual Analog Scale, and functional improvement, assessed through the Western Ontario and McMaster Universities Osteoarthritis Index (WOMAC) score. Additionally, the study will monitor safety and adverse events for up to 12 months after the injection. Secondary outcomes will focus on structural changes in cartilage, evaluated through magnetic resonance imaging (MRI) or X-rays, as well as shifts in synovial inflammatory biomarkers, such as IL-1β, TNF-α, IL-6, and MMP-13. The study aims to determine whether combining the cell-free MSC secretome with Platelet-Rich Growth Factor (PRGF) can alleviate pain, improve joint function, and promote cartilage repair in osteoarthritis (OA). This approach seeks to reduce the risks associated with direct cell transplantation, potentially paving the way for larger trials if the initial results are favorable. The interventional clinical study is expected to be completed by 12 November 2028 [32]. Table 2 summarizes the design, evidence level and transitional stage of each study.

Overall, the registered studies identified in this review demonstrate an increasing interest in translating cell-free therapy based therapeutic approaches to treating musco-skeletal injuries into clinical practice. However, as most of these studies have not yet reported results, their registration should be interpreted as an indication of an emerging research direction rather than as evidence of established clinical efficacy.

Although these clinical trials suggest feasibility and safety, the establishment of standardized products, clearly defined regulatory pathways, GMP-compliant manufacturing and processing, and optimized dosing regimens remain essential for clinical application.

### 3.3. Stem Cell-Derived Extracellular Vesicles (SC-EVs) in Sports Medicine

EVs are nanosized cell-derived products that carry proteins, lipids, and nucleic acids. SC-EVs, which include exosomes and microvesicles, are unique in promoting tissue repair, modulating inflammation, and enhancing regeneration in musculoskeletal injuries [13].

**Figure 3:**
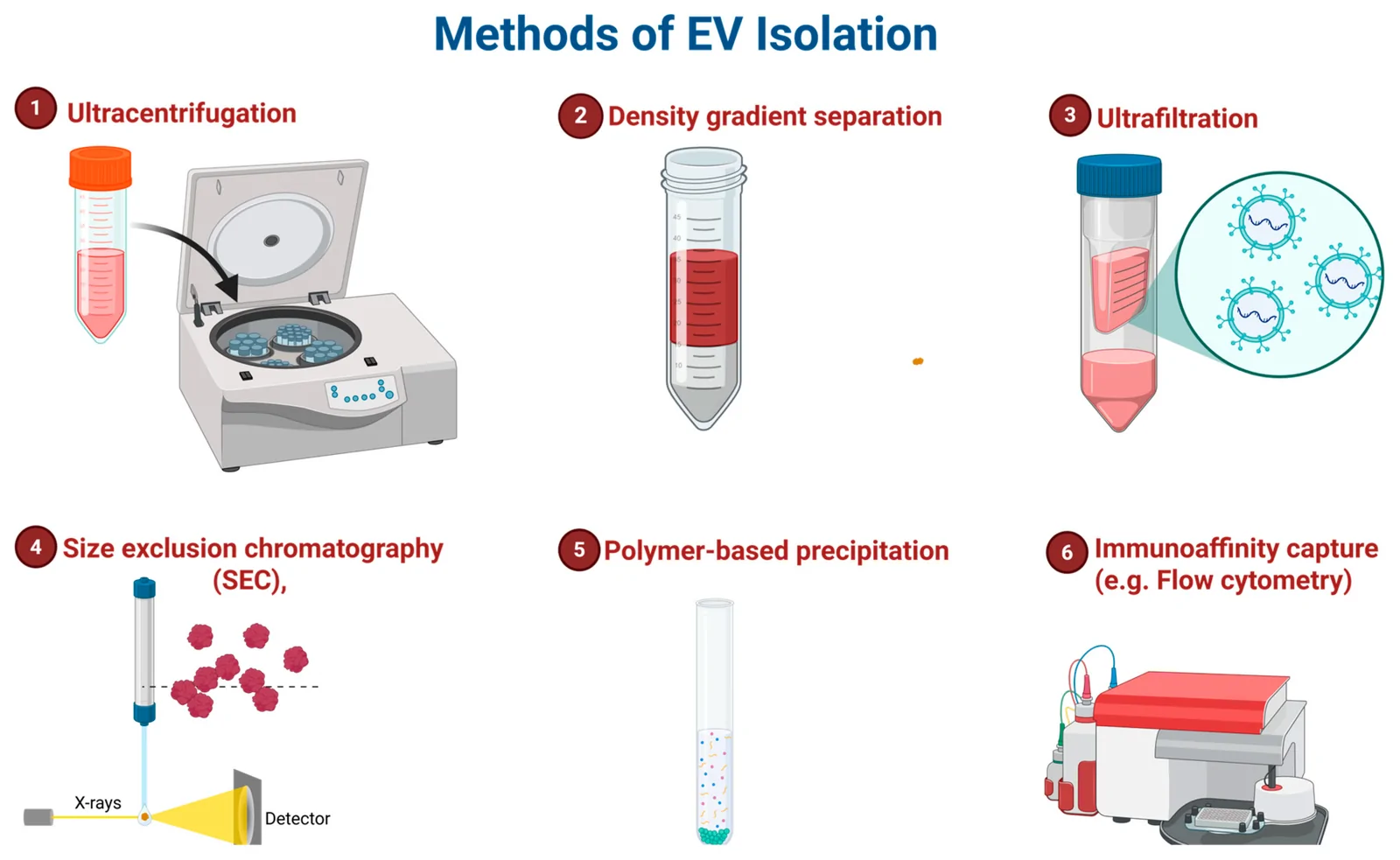
Methods of isolating extracellular vesicles (EVs). Extracellular vesicles (EVs) can be isolated using various methods to process conditioned media for culture or biological fluids. These methods include 1. ultracentrifugation, 2. density gradient separation, 3. ultrafiltration, 4. size exclusion chromatography (SEC), 5. polymer-based precipitation, and 6. immunoaffinity capture. Each of these techniques offers a unique approach to effectively isolate extracellular vesicles.

Stem cell-derived extracellular vesicles (EVs), including exosomes, are extracted from biological fluids or the conditioned media of cell cultures using different extraction methods, as shown in Figure 3. These methods include ultracentrifugation, density gradient separation, ultrafiltration, size exclusion chromatography (SEC), polymer-based precipitation, and immunoaffinity capture, with emerging approaches like material-based biochemical methods and advanced microfluidic or high-purity systems improving yield and purity [33].

Shen and his colleagues studied how EVs produced from adipose-derived stem cells (ASCs) affected the early healing response post tendon injury. The adipose-derived stem cells’ extracellular vesicles (ASC-EVs) were extracted from the conditioned medium of interferon-γ-primed ASCs [34]. These AS-EVs were morphologically characterized and shown to express typical exosome markers before they were applied in a mouse model of Achilles tendon damage, in which a collagen sheet was used at the repair site. NF-κB-luciferase reporter mice were used to monitor inflammation and assess the healing process up to 7 days following surgery. The results showed that the tendon healing rate was significantly increased by increasing NF-κB activity and the expression of pro-inflammatory genes (e.g., Il1b and Ifng). Furthermore, treatment with ASC-EVs from primed ASCs significantly reduced NF-κB activation, pro-inflammatory gene expression, and the development and rupture of repair site tendon gaps. Furthermore, primed ASC-EVs facilitated collagen synthesis at the damage site. The ASC-derived extracellular vesicles (EVs) specifically target infiltrating macrophages and reduce their NF-κB signaling, showcasing their role in immunomodulation. The study demonstrated that ASC-EVs from primed adipose-derived stem cells (ASCs) may enhance tendon healing by modulating the early inflammatory phase and promoting the regeneration of the extracellular matrix. This indicates that ASC-EVs may offer a safer and more effective alternative to direct stem cell transplantation for tendon repair [34]. Shen et al. (2020) [34] investigated the regenerative potential of extracellular vesicles (EVs) released from adipose-derived stem cells (ADSCs) in the healing of Achilles tendons. They extracted stromal cell-derived EVs (SC-EVs) from cultured ADSCs and analyzed their size, shape, and surface markers. The therapeutic effects were assessed through in vitro assays measuring tenocyte proliferation and migration, as well as an in vivo model using rats with Achilles tendon injuries to evaluate tissue regeneration. In vivo studies have shown that treatment with adipose-derived stem cell-derived extracellular vesicles (ADSC-EVs) potentially enhances the proliferation and migration of tenocytes, implying potentially improved collagen fiber alignment, increased deposition of collagen I, reduced fibrosis, and greater biomechanical strength in injured tendons compared to control groups. The research indicates that ADSC-EVs facilitate tendon repair through regenerative and immunomodulatory mechanisms, emphasizing their potential as a cell-free therapy for sports-related tendon injuries. In this study, Shen et al. built upon previous findings by illustrating that ADSC-EVs not only possess significant anti-inflammatory properties but also promote tissue repair by polarizing macrophages to the M2 (pro-repair) phenotype while inhibiting pro-inflammatory cytokines [35].

Shi et al. investigated the potential therapeutic significance of small extracellular vesicles (SC-EVs), primarily exosomes, derived from bone marrow mesenchymal stem cells (BM-MSCs) in the repair of tendons. They extracted EVs from cultured BM-MSCs and examined their size, shape, and expression of specific EV markers. To assess the effects of these EVs, they utilized a mouse model of Achilles tendon damage and conducted additional in vitro experiments with macrophages and tendon cells. The local delivery of BM-MSC-derived EVs was found to significantly enhance tendon healing, which was evidenced by improved collagen fiber alignment, increased deposition of collagen I, reduced scar formation, and superior biomechanical properties compared to the control group [36]. Mesenchymal stem cell-derived extracellular vesicles (MSC-EVs) possess powerful immunomodulatory effects. They inhibit pro-inflammatory cytokines, such as TNF-α and IL-1β, while promoting the polarization of macrophages toward the M2 anti-inflammatory phenotype. This polarization is essential for tissue healing. Overall, this research indicates that extracellular vesicles derived from bone marrow mesenchymal stem cells (BM-MSCs) enhance the important regenerative and anti-inflammatory properties of MSCs. As a result, they hold promise as a cell-free treatment for tendon injuries [36].

A study conducted in 2022 by Song et al. investigated the regenerative potential of exosomes derived from tendon-derived stem cells (TDSCs) in tendon repair. The researchers utilized standard procedures for isolating and characterizing exosomes from cultured TDSCs, which included analyzing size distribution, shape, and surface markers. To examine their therapeutic effects, in vitro experiments were carried out on tenocytes, along with an in vivo tendon injury model. The findings demonstrated that TDSC-derived exosomes may significantly enhance tenocyte proliferation and migration—two critical steps for successful tendon repair. The study identified miR-144-3p as a key component of the exosomal cargo that promotes tendon healing by influencing target genes involved in cell cycle progression and migratory signaling pathways. In the in vivo experiments, treatment with TDSC-exosomes resulted in improved collagen organization, accelerated tissue regeneration, and enhanced structural healing of injured tendons compared to the control group. The study indicates that TDSC-derived exosomes stimulate tendon regeneration through miRNA-mediated modulation of tenocyte behavior, highlighting their potential as a cell-free regenerative therapy for tendon injuries, particularly in the field of sports medicine [37]. Table 2 summarizes the design, evidence level and transitional stage of each research study.

Although MSC-derived EVs have been shown in vitro to regulate inflammatory pathways and stimulate tissue-regenerative processes, these results are primarily mechanistic and should not be taken as proof of clinical efficacy. Similarly, promising outcomes in animal models provide preclinical efficacy evidence but do not prove the safety or efficacy of these treatments in humans.

#### SC-EVs in Clinical Trials

A controlled preclinical study investigated the impact of exosome-educated macrophages (EEMs) and mesenchymal stem cell (MSC)-derived exosomes on recovery from medial collateral ligament (MCL) injuries in rats. The study used human bone marrow-derived MSCs, with the exosomes being identified and characterized before their application. To create EEMs, CD14^+^ macrophages were cultured and educated for three days using high doses of MSC-derived exosomes to promote an anti-inflammatory (M2-like) phenotype. In the in vivo experiments, rats underwent bilateral MCL transection injury. One leg was treated with either EEMs or MSC-derived exosomes, while the other leg received a PBS control treatment. The study employed mechanical tests along with immunohistochemical and histological analyses to evaluate ligament repair after 7 to 14 days. The results indicated that EEM therapy shows potential to enhance ligament mechanical strength, failure load, and maximum stress. Additionally, EEM treatment shifted macrophage populations towards the anti-inflammatory M2 profile, leading to a lower M1/M2 ratio and reduced early inflammation. In contrast, treatment with exosomes alone did not improve mechanical strength or alter macrophage phenotypes. However, it did significantly reduce the size of scar tissue, increase the production of type I and type III collagen, and improve collagen organization in the healing ligament. This suggests enhanced remodeling, albeit without direct effects on tissue strength. These preclinical findings in animals demonstrate that both EEMs and MSC-derived exosomes can aid ligament healing through different mechanisms. EEMs modulate inflammation and strengthen tissue, while exosomes promote extracellular matrix remodeling and reduce scarring. This indicates their potential as therapeutic approaches for ligament injuries, particularly in the field of sports medicine [28].

A Phase I interventional clinical study (NCT06937528), sponsored by the University of Jordan, is currently recruiting participants to investigate the safety and therapeutic potential of injected extracellular vesicles (EVs) for treating knee osteoarthritis (KOA). This study will enroll 50 patients, aged 42 to 75 years and of both genders, who are diagnosed with moderate to severe KOA based on specific inclusion and exclusion criteria. Participants will receive EVs through an intra-articular injection and will be monitored for up to 12 months. This monitoring will include clinical assessments, laboratory investigations, MRI scans of the injected knee, and tracking of any adverse effects. Additionally, preliminary efficacy results will be evaluated, focusing on pain reduction, functional improvement, and quality of life. The primary aim of this clinical study is to assess the safety of EV-based therapies and provide clinical evidence regarding their potential applications in regenerative medicine and inflammation control in osteoarthritis [38].

The clinical trial NCT06466850, sponsored by Isfahan University of Medical Sciences in Iran, is currently recruiting participants for an interventional study. This trial aims to investigate the therapeutic potential of mesenchymal stem cell (MSC)-derived exosomes in patients with knee osteoarthritis (KOA). The study will enroll 20 participants, both male and female, aged between 45 and 75 years, based on specific inclusion and exclusion criteria. Participants will receive intra-articular injections of MSC-derived exosomes on day 1 and again on day 90 in the affected knee joint. Assessments of pain, stiffness, and physical function will be conducted at 1 month, 3 months, and 6 months after treatment using the WOMAC score. The goal of this clinical trial is to determine whether MSC-derived exosomes, which contain bioactive compounds, can effectively regulate inflammation and promote tissue repair, ultimately improving the clinical symptoms and joint function in patients with KOA. The study is expected to be completed by 25 December 2026 [39].

The EXO-OA01 trial NCT06431152 is an open-label Phase 1 pilot clinical study (status: recruiting) sponsored by Universidad de los Andes in Chile. The study aims to assess the safety and feasibility of intra-articular injections of small extracellular vesicles (EVs) or exosomes derived from allogeneic human umbilical cord mesenchymal stem cells (UC-MSC-sEVs) in patients with mild to moderate symptomatic knee osteoarthritis, specifically those with a Kellgren–Lawrence grade of II-III. The 12 participants, aged 30 to 70 with moderate pain (Visual Analog Scale (VAS) ≥ 40 mm), will receive a single intra-articular injection of UC-MSC-sEVs produced according to Good Manufacturing Practice (GMP). The trial employs a dose-escalation approach with three cohorts (low, medium, and high dosages of EVs), each consisting of four patients. Participants are followed for up to 12 months. The primary outcomes of the study focus on safety, toxicity, and feasibility, which include monitoring for adverse reactions, synovitis, and post-injection pain during the initial days and at scheduled follow-up visits. Secondary outcomes were pain levels (analyzed using the Visual Analogue Scale) and function (analyzed through WOMAC scores) after 12 months to determine the appropriate dosage for future trials. This trial is currently ongoing, and as of now, no efficacy data have been reported [40].

**Figure 4:**
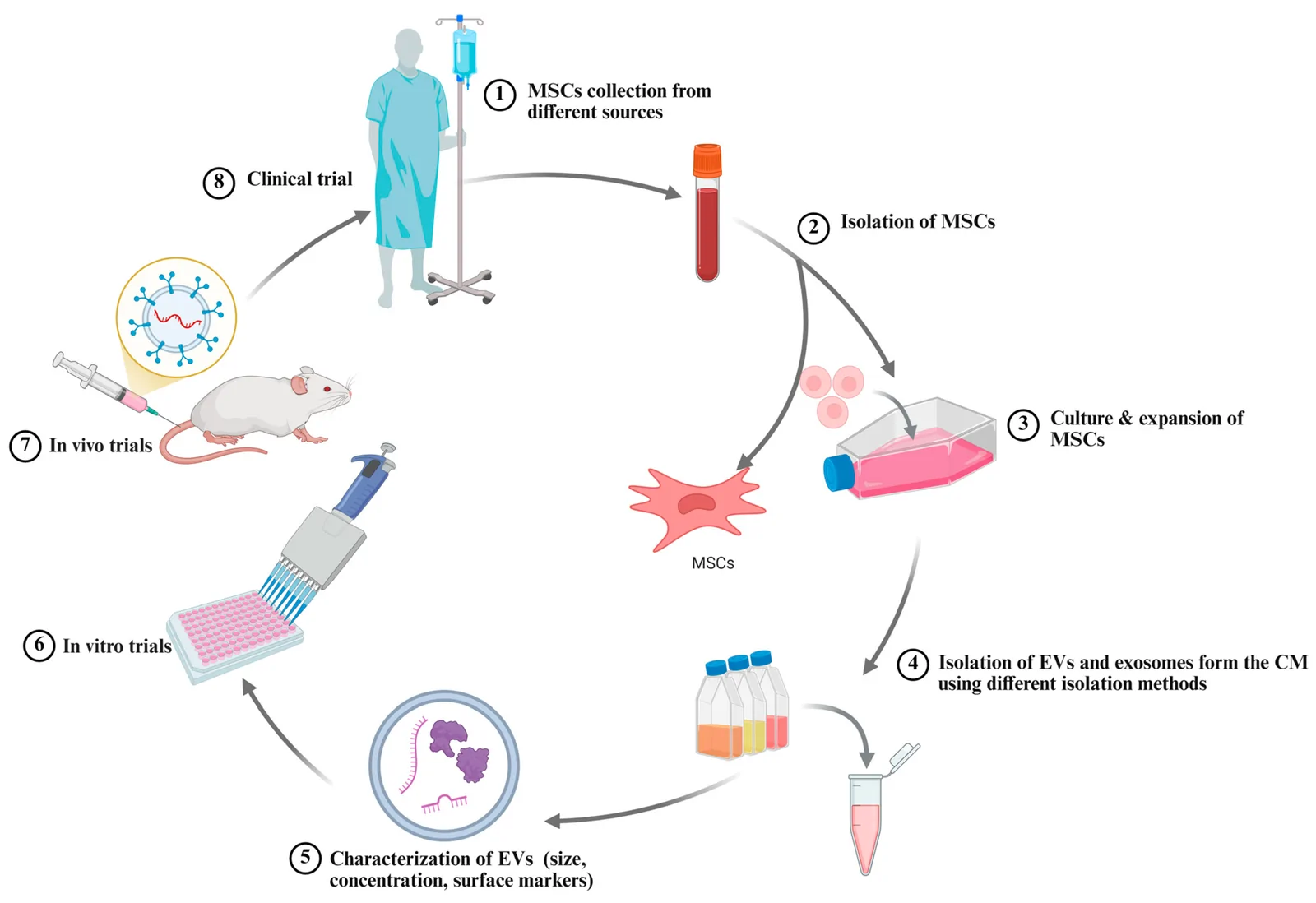
Translational pathway of extracellular vesical (EV)-based therapy. The figure illustrates the progression of mesenchymal stem cell (MSC) secretome- and extracellular vesicle (EV)-based therapies from preclinical to clinical applications. The process involves the collection, isolation, culture and expansion, and isolation of EVs from the conditioned medium (CM) and characterization of the main characteristics. After obtaining the EVs, in vitro, in vivo, and clinical trials are conducted.

Figueroa-Valdés et al. (2025) [41] conducted a study on clinical-grade extracellular vesicles (sEVs) derived from umbilical cord mesenchymal stem cells (UC-MSCs) as a potential intra-articular therapy for knee osteoarthritis (OA). They established a systematic manufacturing procedure to produce batches of clinical-grade sEVs with uniform size, protein, and miRNA profiles, all exceeding regulatory quality standards. In preclinical in vitro studies, UC-MSC sEVs demonstrated biocompatibility and protected human chondrocytes from damage caused by oxidative stress. Additionally, they promoted macrophage polarization toward an anti-inflammatory M2b phenotype through the STAT1 pathway and other mechanisms. In animal models of OA, intra-articular injections of sEVs suggest improved cartilage integrity, reduced bone degeneration, and resulted in better histological scores compared to untreated controls. The sEVs remained localized in the joint and did not migrate to other organs. Further mechanistic analyses indicated that the miRNA and protein cargo of sEVs play roles in regulating macrophages and inflammatory pathways. The research team then conducted a first-in-human clinical trial, injecting UC-MSC-derived sEVs into a patient with moderate knee OA. After a 12-month follow-up, the patient showed improvements in pain and function ratings (measured by VAS and WOMAC), and no adverse events were reported. Imaging studies also revealed that the deterioration of cartilage had stopped. These findings demonstrate the safety, practicality, anti-inflammatory effects, and potential regenerative benefits of UC-MSC-derived EVs for treating knee OA. They also lay the groundwork for Phase I clinical trials to assess dosing and clinical outcomes [41]. However, the majority of the studies that are now accessible as early-phase, pilot, or ongoing clinical trials should not be taken as proof of clinical efficacy. Table 2 summarizes the design, evidence level and transitional stage of each study. Also, Figure 4 illustrates the transitional pathway of EVs from in vitro studies to clinical settings.

**Table 2:** Summary of study design, evidence level, and transitional stage.

Reference	Study Design	Evidence Level	Translational Stage	Main Findings
Soukup et al., 2023 [10]	In vitro tenocyte inflammation model	Preclinical (cell culture)	Early mechanistic investigation	Complete MSC-CM showed greater anti-inflammatory effects compared to the EV fraction alone, indicating the involvement of secreted products greater than EVs.
Lee et al., 2024 [27]	In vitro human tenocyte model under IL-1β-induced tendinopathy conditions	Preclinical (cell culture)	Early mechanistic validation	The CM from SF-UC-MSCs exhibited anti-inflammatory and cytoprotective effects on IL-1β-treated tenocytes from degenerative rotator cuff injuries.
Chamberlain et al., 2021 [28]	Animal study (Ligament injury model)	Preclinical (animal)	Preclinical translational stage	Exosomes and exosome-derived macrophages enhanced ligament healing and regulated immunological responses.
Chen et al., 2019 [29]	Animal study (Rat model)	Preclinical (animal)	Preclinical efficacy evaluation	MSC-CM mitigated OA progression by conserving subchondral bone, maintaining matrix integrity, and promoting autophagy.
Zhang et al., 2021 [30]	Animal study (Achilles tendon injury model)	Preclinical (animal)	Preclinical efficacy evaluation	Tendon stem cell-derived CM facilitated Achilles tendon healing through regenerative processes associated with hepatocyte growth factor signaling.
BioCorTherapies, NCT05777213 [31]	Clinical trial (registered)	Clinical (ongoing)	Early clinical investigation	The clinical trial is still ongoing; no findings yet
University of Punjab, NCT07157891 [32]	Clinical trial (registered)	Clinical (ongoing)	Early clinical investigation	The clinical trial is still ongoing; no findings yet
Shen et al., 2020 [34]	Animal study (Tendon injury model)	Preclinical (animal)	Preclinical translational stage	EVs of ADSCs inhibited early inflammation and improved tendon healing outcomes.
Chen et al., 2021 [35]	Animal study (Achilles tendon injury model)	Preclinical (animal)	Preclinical efficacy evaluation	ADSC-derived EVs enhanced recovery of the injured Achilles tendons.
Shi et al., 2019 [36]	Animal study	Preclinical (animal)	Preclinical translational stage	BM-MSC-derived EVs modulated inflammation and promoted tendon regeneration.
Song et al., 2022 [37]	In vitro + animal study	Preclinical (cell culture + animal)	Mechanistic and preclinical validation	Exosomes improved tendon repair via miR-144-3p-mediated modulation of tenocyte proliferation and migration.
University of Jordan, NCT06937528 [38]	Clinical trial (registered)	Clinical (ongoing)	Early clinical investigation	The clinical trial is still ongoing; no findings yet
Isfahan University, NCT06466850 [39]	Clinical trial (registered)	Clinical (ongoing)	Early clinical investigation	The clinical trial is still ongoing; no findings yet
Universidad de los Andes, EXO-OA01 (NCT06431152) [40]	Clinical trial (registered)	Clinical (ongoing)	Early clinical investigation	The clinical trial is still ongoing; no findings yet
Figueroa-Valdés et al., 2025 [41]	Preclinical development + first-in-human validation	Translational + early clinical	First-in-human clinical stage	The clinical trial is at the first-in human-clinical stage; no findings yet

## 4. Limitations of Stem Cell-Derived Products in Sports Medicine

Standardization and reproducibility pose significant challenges in the use of stem cells in sports medicine. Extracellular vesicles (EVs) and conditioned media (CMs) are complex mixtures containing proteins, lipids, nucleic acids, and other bioactive substances. Their composition varies widely depending on factors such as the source of stem cells, donor characteristics, culture conditions, and isolation procedures. This unpredictability complicates the production of consistent batches with reliable therapeutic effects, which affects both experimental comparisons and regulatory approval processes. Unlike conventional medications, there are no globally accepted guidelines defining an effective or potent secretome product. Furthermore, variations in culture medium, cell passage numbers, and induction settings can lead to inconsistent findings. Additionally, the pharmacokinetics of EVs and CMs remain insufficiently understood. Unlike small-molecule medications, these substances lack established dose–response curves, half-lives, and optimal delivery routes for human subjects. Although preclinical cell culture studies demonstrate biological activity, it remains unclear how these findings translate into effective doses for humans. The rapid clearance of these substances from circulation may further limit their therapeutic efficacy [10]. Furthermore, differences in cell source, donor variability, culture conditions, isolation procedures, and product characterization limit repeatability and make direct comparisons among research studies difficult. This limitation agrees with the new MISEV2023 guidelines, which emphasize thorough EV definition, characterization, and reporting [8]. Moreover, the majority of published studies are limited to in vitro experiments or small-animal models with short follow-up periods, making long-term tissue remodeling, biomechanical recovery, and safety implications difficult to conclude. Recent studies of MSC secretome- and EV-based therapeutics emphasize the necessity for consistent manufacturing, appropriate dose, and well-designed clinical trials before these methods can be implemented into clinical practice [9]. Given the variance in the level of evidence and translational stage, this paper presents a narrative review rather than a systemic review. and No formal risk-of-bias assessment was performed, but the included studies were classified based on their level of evidence using study design.

## 5. Conclusions

In conclusion, stem cell-derived products, such as extracellular vesicles (EVs) and conditioned media (CMs), represent a promising new frontier in sports medicine. They offer cell-free methods for improving musculoskeletal repair and modulating inflammation. Preclinical evidence indicates that these products can enhance the repair of tendons, ligaments, and cartilage. Preliminary clinical studies also show promising safety profiles and potential for regeneration. However, further research and standardized protocols are necessary to fully unlock the therapeutic potential of these products as current evidence is mainly preclinical, with the majority of findings based on in vitro and animal research, and clinical evidence is still very limited. As a result, the therapeutic efficacy of these approaches has yet to be fully determined. Future research should focus on well-designed, correctly powered randomized controlled clinical trials with long-term follow-up, as well as the development of standardized protocols for EV and CM separation, characterization, quality control, dosage assessment, and delivery techniques. The ultimate goal of addressing these challenges is to provide safe and effective regenerative treatments that accelerate recovery and reduce the risk of reinjury for athletes.

## Figures and Tables

**Table 1:** Variability aspects, variation across studies, and the potential impact of stem cell-derived conditioned media (SC-CM) in sports medicine.

Variability Aspect	Variations Across Studies	Potential Impact
Source of MSCs	MSCs can be isolated from various tissue sources including the bone marrow, adipose tissue, umbilical cord, and tendon related-tissue [18,24].	Different sources of MSCs may influence the EVs’ cargo composition and their biological effect, resulting in biologically non-bioequivalent EV products [16,18].
Culture conditions of MSCs	Cell culture conditions, donor demographics and health status, cellular environment, and preconditioning or licensing approaches [10,23].	The variation in culture conditions may affect the secretory activity (different secretome composition), EV production, cargo composition and, consequently, the therapeutic function [10,23].
EV isolation & purification	EVs can be isolated using ultracentrifugation, size-exclusion chromatography, precipitation-based methods, and other emerging methods [24,30].	Variation in isolation methods can affect the EV yield, purity, and the presence of non-EV components, limiting the consistency between different preparations [24,30].
EV characterization	Characterization methods can vary in the assessment of EV morphology, size, concentration, molecular cargo, and EV markers [16,18].	Inconsistent EVs characterization prevents equal comparison between preparations [24,31].
Dosing and dose reporting	Therapeutic doses can be reported using several parameters, such as particle number, protein concentration, administered volume, or other product-specific measurements [24,30].	Non-defined dosing parameters limit the dose-response comparison and the identification of the optimal therapeutic dose [24,30].
Delivery route	Therapeutic products may be administered through different routes, including intra-articular, intravenous, local, or topical administration [18,24].	Differences in delivery route may influence biodistribution, tissue exposure, retention, and therapeutic efficacy [18,24].

## Data Availability

No new data were created or analyzed in this study. Data sharing is not applicable to this article.

## Abbreviations

The following abbreviations are used in this manuscript:

Abbreviation	Definition
MSCs	Mesenchymal Stem Cells
EVs	Extracellular Vesicles
CM	Conditioned Medium
OA	Osteoarthritis
BM-MSCs	Bone Marrow-Derived Mesenchymal Stem Cells
UC-MSCs	Umbilical Cord-Derived Mesenchymal Stem Cells
ASCs	Adipose-Derived Stem Cells
TDSCs	Tendon-Derived Stem Cells
TSCs	Tendon Stem Cells
PBMCs	Peripheral Blood Mononuclear Cells
PBS	Phosphate-Buffered Saline
GMP	Good Manufacturing Practice
MRI	Magnetic Resonance Imaging
VAS	Visual Analog Scale
WOMAC	Western Ontario and McMaster Universities Osteoarthritis Index
PRGF	Plasma Rich in Growth Factors
IL	Interleukin
TNF-α	Tumor Necrosis Factor Alpha
COX-2	Cyclooxygenase-2
MMPs	Matrix Metalloproteinases
TIMP	Tissue Inhibitor of Metalloproteinases
TGF-β	Transforming Growth Factor Beta
IGF-1	Insulin-Like Growth Factor 1
bFGF	Basic Fibroblast Growth Factor
HGF	Hepatocyte Growth Factor
NF-κB	Nuclear Factor Kappa B
LC3	Microtubule-Associated Protein 1 Light Chain 3
SEC	Size Exclusion Chromatography
DCs	Dendritic Cells
Treg	Regulatory T Cells
miRNA	MicroRNA

## References

[1] Yang S.X., Cheng S., Su D.L. (2022). Sports injury and stressor-related disorder in competitive athletes: A systematic review and a new framework. Burns Trauma.

[2] Demeco A., De Sire A., Marotta N., Spanò R., Lippi L., Palumbo A., Iona T., Gramigna V., Palermi S., Leigheb M. (2022). Match analysis, physical training, risk of injury and rehabilitation in padel: Overview of the literature. Int. J. Environ. Res. Public Health.

[3] Lambert C., Ritzmann R., Akoto R., Lambert M., Pfeiffer T., Wolfarth B., Lachmann D., Shafizadeh S. (2022). Epidemiology of injuries in Olympic sports. Int. J. Sports Med..

[4] Torvaldsson K., Lindblom H., Sonesson S., Senorski E.H., Stigson H., Tamm L., Sandberg J., Hägglund M. (2023). Swedish Olympic athletes report one injury insurance claim every second year: A 22-year insurance registry-based cohort study. Knee Surg. Sports Traumatol. Arthrosc..

[5] Trebinjac S., Gharairi M. (2020). Mesenchymal stem cells for treatment of tendon and ligament injuries-clinical evidence. Med. Arch..

[6] Lahuerta-Martin S., Robles-Perez R., Hernando-Garijo I., Jiménez-Del-Barrio S., Hernández-Lázaro H., Mingo-Gómez M.T., Ceballos-Laita L. (2023). The effectiveness of non-surgical interventions in athletes with groin pain: A systematic review and meta-analysis. BMC Sports Sci. Med. Rehabil..

[7] Stewart C.E. (2021). Stem cells and regenerative medicine in sport science. Emerg. Top. Life Sci..

[8] Zumwalt M., Reddy A.P. (2020). Stem cells for treatment of musculoskeletal conditions-orthopaedic/sports medicine applications. Biochim. Biophys. Acta (BBA)-Mol. Basis Dis..

[9] Palermi S., Gnasso R., Belviso I., Iommazzo I., Vecchiato M., Marchini A., Corsini A., Vittadini F., Demeco A., De Luca M. (2023). Stem cell therapy in sports medicine: Current applications, challenges and future perspectives. J. Basic Clin. Physiol. Pharmacol..

[10] Soukup R., Gerner I., Mohr T., Gueltekin S., Grillari J., Jenner F. (2023). Mesenchymal stem cell conditioned medium modulates inflammation in tenocytes: Complete conditioned medium has superior therapeutic efficacy than its extracellular vesicle fraction. Int. J. Mol. Sci..

[11] Jung H., Jung Y., Seo J., Bae Y., Kim H.-S., Jeong W. (2024). Roles of extracellular vesicles from mesenchymal stem cells in regeneration. Mol. Cells.

[12] Phinney D.G., Pittenger M.F. (2017). Concise review: MSC-derived exosomes for cell-free therapy. Stem Cells.

[13] Aziziyan F., Asl S.S., Mahdipour M., Fard R.N., Sheykhhasan M. (2026). Mesenchymal stem cell-derived extracellular vesicles in musculoskeletal regeneration: Mechanisms, applications, and future prospects. Stem Cell Res. Ther..

[14] Ocansey D.K., Zhang L., Wang Y., Yan Y., Qian H., Zhang X., Xu W., Mao F. (2020). Exosome-mediated effects and applications in inflammatory bowel disease. Biol. Rev..

[15] Ma Z.-J., Yang J.-J., Lu Y.-B., Liu Z.-Y., Wang X.-X. (2020). Mesenchymal stem cell-derived exosomes: Toward cell-free therapeutic strategies in regenerative medicine. World J. Stem Cells.

[16] Deng H., Sun C., Sun Y., Li H., Yang L., Wu D., Gao Q., Jiang X. (2018). Lipid, protein, and microRNA composition within mesenchymal stem cell-derived exosomes. Cell. Reprogramming.

[17] Foo J.B., Looi Q.H., How C.W., Lee S.H., Al-Masawa M.E., Chong P.P., Law J.X. (2021). Mesenchymal stem cell-derived exosomes and MicroRNAs in cartilage regeneration: Biogenesis, efficacy, miRNA enrichment and delivery. Pharmaceuticals.

[18] Wang Z.-G., He Z.-Y., Liang S., Yang Q., Cheng P., Chen A.-M. (2020). Comprehensive proteomic analysis of exosomes derived from human bone marrow, adipose tissue, and umbilical cord mesenchymal stem cells. Stem Cell Res. Ther..

[19] Donoso-Quezada J., Ayala-Mar S., González-Valdez J. (2021). The role of lipids in exosome biology and intercellular communication: Function, analytics and applications. Traffic.

[20] Lo Sicco C., Reverberi D., Balbi C., Ulivi V., Principi E., Pascucci L., Becherini P., Bosco M.C., Varesio L., Franzin C. (2017). Mesenchymal stem cell-derived extracellular vesicles as mediators of anti-inflammatory effects: Endorsement of macrophage polarization. Stem Cells Transl. Med..

[21] Jung S., Lee S., Kim H.J., Kim S., Moon J.H., Chung H., Kang S.-J., Park C.-G. (2023). Mesenchymal stem cell-derived extracellular vesicles subvert Th17 cells by destabilizing RORγt through posttranslational modification. Exp. Mol. Med..

[22] Shahir M., Mahmoud Hashemi S., Asadirad A., Varahram M., Kazempour-Dizaji M., Folkerts G., Garssen J., Adcock I., Mortaz E. (2020). Effect of mesenchymal stem cell-derived exosomes on the induction of mouse tolerogenic dendritic cells. J. Cell. Physiol..

[23] Wang J., Moosavizadeh S., Jammes M., Tabasi A., Bach T., Ryan A.E., Ritter T. (2025). In-vitro immunomodulatory efficacy of extracellular vesicles derived from TGF-β1/IFN-γ dual licensed human bone marrow mesenchymal stromal cells. Stem Cell Res. Ther..

[24] Eshraghi N., Javidan A., Al-Saeidi N.N., Makuku R., Mortezaei A., Mirghaderi P. (2025). Mesenchymal Stem Cell–Derived Exosome Efficacy and Safety in Musculoskeletal Tissues: State of The Art and Future Directions. Regen. Eng. Transl. Med..

[25] Song X., Liu F., Chen M., Zhu M., Zheng H., Wang W., Chen D., Li M., Chen S. (2024). MiR-21 regulates skeletal muscle atrophy and fibrosis by targeting TGF-beta/SMAD7-SMAD2/3 signaling pathway. Heliyon.

[26] Gunawardena T.N.A., Rahman M.T., Abdullah B.J.J., Abu Kasim N.H. (2019). Conditioned media derived from mesenchymal stem cell cultures: The next generation for regenerative medicine. J. Tissue Eng. Regen. Med..

[27] Lee M.J., Park K., Yeon Lee S., Jang K.-H., Won S., Jo C.H. (2024). Effects of Conditioned Media From Human Umbilical Cord-Derived Mesenchymal Stem Cells on Tenocytes From Degenerative Rotator Cuff Tears in an Interleukin 1β–Induced Tendinopathic Condition. Orthop. J. Sports Med..

[28] Chamberlain C.S., Kink J.A., Wildenauer L.A., McCaughey M., Henry K., Spiker A.M., Halanski M.A., Hematti P., Vanderby R. (2021). Exosome-educated macrophages and exosomes differentially improve ligament healing. Stem Cells.

[29] Chen W., Sun Y., Gu X., Hao Y., Liu X., Lin J., Chen J., Chen S. (2019). Conditioned medium of mesenchymal stem cells delays osteoarthritis progression in a rat model by protecting subchondral bone, maintaining matrix homeostasis, and enhancing autophagy. J. Tissue Eng. Regen. Med..

[30] Zhang Z., Li Y., Zhang T., Shi M., Song X., Yang S., Liu H., Zhang M., Cui Q., Li Z. (2021). Hepatocyte growth factor-induced tendon stem cell conditioned medium promotes healing of injured Achilles tendon. Front. Cell Dev. Biol..

[31] BioCorTherapies Potential Injection of Human Umbilical Cord Secretome in Trophic Ulcers Treatment (NCT05777213). NCT05777213.

[32] UniversityofthePunjab Investigating the Safety and Regenerative Potential of MSC-Derived Secretome Combined with PRGF in Knee Osteoarthritis (NCT07157891). NCT07157891.

[33] Chen M., Li J., Lin Y., Li X., Yu Y., Zhou S., Xu F., Zhang Q., Zhang H., Wang W. (2024). Recent research on material-based methods for isolation of extracellular vesicles. Anal. Methods.

[34] Shen H., Yoneda S., Abu-Amer Y., Guilak F., Gelberman R.H. (2020). Stem cell-derived extracellular vesicles attenuate the early inflammatory response after tendon injury and repair. J. Orthop. Res..

[35] Chen S.-H., Chen Z.-Y., Lin Y.-H., Chen S.-H., Chou P.-Y., Kao H.-K., Lin F.-H. (2021). Extracellular vesicles of adipose-derived stem cells promote the healing of traumatized achilles tendons. Int. J. Mol. Sci..

[36] Shi Z., Wang Q., Jiang D. (2019). Extracellular vesicles from bone marrow-derived multipotent mesenchymal stromal cells regulate inflammation and enhance tendon healing. J. Transl. Med..

[37] Song K., Jiang T., Pan P., Yao Y., Jiang Q. (2022). Exosomes from tendon derived stem cells promote tendon repair through miR-144-3p-regulated tenocyte proliferation and migration. Stem Cell Res. Ther..

[38] University of Jordan Use of Extracellular Vesicles (EV) for Knee Osteoarthrosis (NCT06937528). NCT06937528.

[39] Isfahan University of Medical Sciences Mesenchymal Stem Cells Derived Exosomes in Osteoarthritis Patients (NCT06466850). NCT06466850.

[40] Universidad de los Andes C Intra-articular Injection of UC-MSC Exosome in Knee Osteoarthritis (EXO-OA01). https://clinicaltrials.gov/ct2/show/NCT06431152.

[41] Figueroa-Valdés A.I., Luz-Crawford P., Herrera-Luna Y., Georges-Calderón N., García C., Tobar H.E., Araya M.J., Matas J., Donoso-Meneses D., de la Fuente C. (2025). Clinical-grade extracellular vesicles derived from umbilical cord mesenchymal stromal cells: Preclinical development and first-in-human intra-articular validation as therapeutics for knee osteoarthritis. J. Nanobiotechnology.

